# Taxonomy Portraits: Deciphering the Hierarchical Relationships of Medical Large Language Models

**DOI:** 10.2196/72918

**Published:** 2025-10-07

**Authors:** Radha Nagarajan, Vanessa Klotzman, Midori Kondo, Sandip Godambe, Adam Gold, John Henderson, Steven Martel

**Affiliations:** 1 Rady Children's Health, 1201 W La Veta Ave, Orange, CA, 92868, United States, 1 714-997-3000; 2 Fred Hutch Cancer Center, Seattle, WA, United States

**Keywords:** large language models, health care, performance benchmark, taxonomy, redundancy

## Abstract

**Background:**

Large language models (LLMs) continue to enjoy enterprise-wide adoption in health care while evolving in number, size, complexity, cost, and most importantly performance. Performance benchmarks play a critical role in their ranking across community leaderboards and subsequent adoption.

**Objective:**

Given the small operating margins of health care organizations and growing interest in LLMs and conversational artificial intelligence (AI), there is an urgent need for objective approaches that can assist in identifying viable LLMs without compromising their performance. The objective of the present study is to generate taxonomy portraits of medical LLMs (n=33) whose domain-specific and domain non-specific multivariate performance benchmarks were available from Open-Medical LLM and Open LLM leaderboards on Hugging Face.

**Methods:**

Hierarchical clustering of multivariate performance benchmarks is used to generate taxonomy portraits revealing inherent partitioning of the medical LLMs across diverse tasks. While domain-specific taxonomy is generated using nine performance benchmarks related to medicine from the Hugging Face Open-Medical LLM initiative, domain non-specific taxonomy is presented in tandem to assess their performance on a set of six benchmarks and generic tasks from the Hugging Face Open LLM initiative. Subsequently, non-parametric Wilcoxon rank-sum test and linear correlation are used to assess differential changes in the performance benchmarks between two broad groups of LLMs and potential redundancies between the benchmarks.

**Results:**

Two broad families of LLMs with statistically significant differences (α=.05) in performance benchmarks are identified for each of the taxonomies. Consensus in their performance on the domain-specific and domain non-specific tasks revealed robustness of these LLMs across diverse tasks. Subsequently, statistically significant correlations between performance benchmarks revealed redundancies, indicating that a subset of these benchmarks may be sufficient in assessing the domain-specific performance of medical LLMs.

**Conclusions:**

Understanding medical LLM taxonomies is an important step in identifying LLMs with similar performance while aligning with the needs, economics, and other demands of health care organizations. While the focus of the present study is on a subset of medical LLMs from the Hugging Face initiative, enhanced transparency of performance benchmarks and economics across a larger family of medical LLMs is needed to generate more comprehensive taxonomy portraits for accelerating their strategic and equitable adoption in health care.

## Introduction

Large language models (LLMs) continue to show considerable promise and growth in health care [1]. Popular health care LLM applications fall under three broad task categories, namely clinical tasks, documentation tasks, and medical research and education tasks [2]. Specific LLM health care applications include (1) virtual health assistants and language translation [3], (2) summarization of clinical narratives and ambient listening [4,5], (3) patient education [6], and (4) clinical trial matching [7]. More importantly, LLMs have continued to evolve in numbers, size, complexity, costs, and performance, impacting their adoption [8]. A recent perspective discussed three broad LLM implementation pathways (Training from Scratch, Fine-Tuned Pathway, and Out of the Box Pathway) along with the risks, benefits, and economics across four major cloud service providers for their equitable and strategic adoption in health care [9]. The study also elucidated the essential ingredients such as digital data, infrastructure, workforce, ethics, and regulatory aspects that can significantly impact LLM implementations. While helpful, these three pathways represent broad categorizations of LLM implementations and do not necessarily provide insights into their similarities. Similarities between LLMs can be based on a number of characteristics including architecture, size, cost, and their performance across diverse tasks [10]. Understanding the similarities in LLM performance can assist in strategically selecting those with comparable performance while aligning with the budgeting and needs of health care organizations.

The present study focuses on standardized and objective performance benchmarks that interrogate the ability of LLMs across diverse tasks. Their weighted average, is often used to rank LLMs across leaderboards [11], impacting their adoption. These aggregated benchmarks implicitly map the multivariate benchmark profiles onto a univariate score, diminishing their usefulness, as each benchmark interrogates unique capabilities of the LLMs. Therefore, it should not be surprising to note that similarity in ranks may not necessarily imply similarity in performance benchmark profiles. The present study generates LLM taxonomies elucidating their similarities and hierarchical associations from multivariate performance benchmarks. The taxonomy is shown to reveal inherent partitioning of the LLMs into sub-groups with varying performance. LLMs can be either open-source or closed source. While these implementations have distinct advantages [12], proprietary aspects and lack of transparency in the performance of closed-source LLMs prevent their inclusion in the present study. In the case of open-source LLMs, domain-specific (DS) as well as domain non-specific (DN) multivariate performance benchmarks were available publicly from Open LLM [13] and Open Medical LLM [14] leaderboards at Hugging Face [15,16]. While DS benchmarks interrogate task-specific abilities of the LLMs, DN benchmarks assess their generic capabilities. Hugging Face has witnessed increasing visibility, growth, and adoption by the Generative AI and LLM communities over the years. Its structured and transparent approach enables enhanced reproducibility of the reported metrics and implementation; widespread collaboration between experts; unbiased comparison of the different models; and the selection of the LLMs based on the performance, needs, and affordability. The DS benchmarks considered include those that assess the medical question and answering capabilities and reasoning skills related to medical licensing examinations, a series of subject and DS evaluations broadly under massive multitask language understanding, and the ability of LLMs to comprehend and reason biomedical literature. The DN benchmarks were also retrieved for the medical LLMs through the Open LLM initiative to assess their ability to answer questions that are not specifically related to medical tasks. These benchmarks included (1) those that assess the LLMs ability to follow verifiable instructions, (2) chain of thought prompting, (3) mathematical problem-solving skills, (4) graduate level reasoning capabilities across diverse subjects, (5) multistep reasoning abilities, and (6) multitask language understanding on challenging reasoning-based questions. A detailed description of the DS and DN benchmarks along with the references and their abbreviations is included in Table 1. The taxonomies were generated by hierarchical clustering of the DS and DN multivariate performance benchmarks that assess the task-specific and generic capabilities of these LLMs. Subsequently, two broad groups of medical LLMs with markedly different performance benchmark profiles is discussed. Potential redundancies between the performance benchmarks across the DS and DN taxonomies are also elucidated. Given the low-operating margins [17] of health care organizations, understanding the taxonomy and potential redundancies between the performance benchmarks is expected to assist in objectively justifying the choice of LLMs while controlling costs [18].

**Table 1.** Description and abbreviations of domain-specific (DS) and domain non-specific (DN) performance benchmarks with references.

Type	Benchmark description	Abbreviation
DS	MedQA [Medical Question and Answer]: Consists of multiple-choice questions (11,450 questions in the development set and 1273 questions in the test set) from the United States Medical License Exam for benchmarking the LLMs general medical knowledge and reasoning skills on United States Medical Licensure.	MQA [19**]**
DS	MedMCQA [Medical Multiple-Choice Question and Answer]: Consists of multiple-choice questions (187,000 questions in the development set and 6100 questions in the test set) related to the Indian Medical Entrance Exam (AIIMS/NEET). As with MedQA, MedMCQA is used to benchmark the LLMs general medical knowledge and reasoning ability as it pertains to the Indian medical entrance exam.	MCQA [20**]**
DS	MMLU Anatomy: [Massive Multitask Language Understanding, Anatomy]: MMLU subset consists of multiple-choice questions (135 questions) for benchmarking the knowledge of the LLM^a^ on human anatomy.	ANAT [21**]**
DS	MMLU Clinical Knowledge [Massive Multitask Language Understanding, Clinical Knowledge]: MMLU subset consists of multiple-choice questions (265 questions) for benchmarking the clinical knowledge and decision-making skills.	CLIN [21**]**
DS	MMLU College Biology [Massive Multitask Language Understanding, College Biology]: MMLU subset with multiple-choice questions (144 questions) for benchmarking the knowledge on college biology.	BIOL [21**]**
DS	MMLU College Medicine [Massive Multitask Language Understanding, College Medicine] MMLU subset with multiple-choice questions (173 questions) for benchmarking the college-level medical knowledge.	CMED [21**]**
DS	MMLU Medical Genetics [Massive Multitask Language Understanding, Medical Genetics] MMLU subset consists of 100 questions related to medical genetics.	GEN [21**]**
DS	MMLU Professional Medicine [Massive Multitask Language Understanding, Professional Medicine] MMLU subset consists of multiple-choice questions (272 questions) for benchmarking the LLM on knowledge required for medical professionals.	PMED [21**]**
DS	PUBMEDQA [PUBMED Question & Answer] Closed-domain dataset comprising expert-labeled question-answer pairs (500 questions in the development set and 500 questions in the test set) for benchmarking the LLMs ability to comprehend and reason biomedical literature.	PUBM [22**]**
DN	IFEval [Instruction Following Evaluation]: Benchmarks LLMs ability to follow verifiable instructions using 25 distinct types of verifiable instructions and 500 prompts, with each prompt containing at least one verifiable instruction.	IFEV [23**]**
DN	BBH [Big Bench Hard]: Benchmarks the performance of LLMs on 23 challenging Big Bench tasks (BBH) where prior LLMs failed to outperform an average-human rater. Emphasized the importance of chain-of-thought prompting.	BBH [24**]**
DN	MATH [Math]: Benchmarks the mathematical problem-solving ability of the LLM using 12,500 mathematics competition problems.	MATH [25**]**
DN	GPQA [Graduate Level Google Proof Q & A]: Benchmarks LLMs using 448 multiple choice questions generated by experts in areas such as biology, physics, and chemistry.	GPQA [26**]**
DN	MuSR [Multistep Soft Reasoning]: Benchmarks LLMs ability on complex multistep reasoning instances and long-range (~1000 words) free text narratives from real-world domains.	MUSR [27**]**
DN	MMLU Pro [Multitask Language Understanding Pro]: Benchmarks the reasoning and language comprehension abilities of LLMs across diverse domains by incorporating challenging, reasoning-focused question, and expanding the choice of the original MMLU from four to ten.	MPRO [28**]**

a LLM: large language model.

## Methods

Medical LLMs with DS and DN benchmarks were retrieved from Hugging Face Open Medical LLM [14] and Open LLM [13] leaderboards on January 2025. While Hugging Face features several contributions from the AI open-source community, it is important to note that these are voluntary efforts. Since there were instances of sparse documentation across LLMs by individual contributors, the present study excluded LLMs by individual contributors, resulting in 33 medical LLMs whose DS and DN performance benchmarks were available. Abbreviations and size of the 33 LLMs are enclosed in Table 2. LLM taxonomies were generated using hierarchical clustering [29] of the DS and DN multivariate performance benchmarks. As each performance benchmark interrogates specific characteristic of the LLM, they were scaled to zero-mean and unit variance prior to clustering to minimize the impact of potential variations in the magnitude across the different benchmarks. Subsequently, the Manhattan distance was used to assess the similarity between the LLMs, as it is robust to outliers. Other measures of similarity such as cosine distance can also be used as alternatives [29]. This study uses complete linkage that merges clusters based on the distance between the most dissimilar members (ie, farthest distance) [29]. While complete linkage is robust to outliers resulting in stable and well-formed clusters, other linkage approaches for merging the clusters can also be explored [29].

The DS taxonomy was based on the nine performance benchmarks, whereas the DN taxonomy was generated based on the six performance benchmarks, as shown in Table 1. Color-coded dendrograms were subsequently used to generate visualizations of the performance benchmark profiles of the respective taxonomies. Statistically significant differential changes in performance benchmark profiles between clusters for the DS and DN taxonomy were investigated using the Wilcoxon rank-sum test (α=.01), a non-parametric statistical test that does not impose normality assumptions on their distribution. Subsequently, Pearson correlation and scatter plots were used to elucidate potential redundancies between the performance benchmarks for the DS and DN taxonomies. Pearson correlation can provide insights into linear dependency (a=0.01) between variables. However, its estimates can be deceptive under sparse distribution of data points about the linear trend. Therefore, scatter plots of the pair-wise performance benchmarks are provided in addition to the statistical test for visualization. As differential changes in the performance benchmarks and the test for correlation involved multiple statistical tests, multiple testing correction (Bonferroni correction) [30] was used to control for the family-wise error rate, with the adjusted significance level α^*^ given by α^*^= α/M, where M represents the total number of statistical tests.

**Table 2.** Open-source large language models (LLMs; n=33) from Hugging Face with their abbreviations.

Open-source LLM (Hugging Face)	Abbreviation	Open-source LLM (contd.)	Abbreviation (contd.)
mistralai/Mistral-7B-Instruct-v0.1	MISI-7B^a^	VAGOsolutions/SauerkrautLM-Gemma-7b	GEMS-7B
mistralai/Mistral-7B-v0.1	MIS-7B	VAGOsolutions/Llama-3-SauerkrautLM-8b-Instruct	LM3SI-8B
EleutherAI/pythia-2.8b	PYT-2.8B	openai-community/gpt2-xl	GPTL-1.5B
EleutherAI/gpt-neo-2.7B	GPTN-2.7B	openai-community/gpt2	GPT2-1.5B
lmsys/vicuna-7b-v1.5	VIC-7B	HuggingFaceH4/zephyr-7b-beta	ZEP-7B
abacusai/Llama-3-Smaug-8B	LM3S-8B	tiiuae/falcon-7b-instruct	FALI-7B
abacusai/Liberated-Qwen1.5-14B	QWN-14B	tiiuae/falcon-7b	FAL-7B
HPAI-BSC/Llama3-Aloe-8B-Alpha	LM3A-8B	NousResearch/Nous-Hermes-2-Mistral-7B-DPO	MISD-7B
google/gemma-2b	GEM-2B	NousResearch/Hermes-2-Pro-Mistral-7B	MISH-7B
google/gemma-1.1-7b-it	GEMI-7B	CohereForAI/aya-23-8B	AYA-8B
google/recurrentgemma-2b	GEMR-2B	upstage/SOLAR-10.7B-Instruct-v1.0	SL-10.7B
google/gemma-7b	GEM-7B	01-ai/Yi-1.5-9B-32K	YIK-9B
microsoft/phi-1_5	PHI-1.3B	01-ai/Yi-1.5-9B	YI-9B
TinyLlama/TinyLlama-1.1B-intermediate-step-1431k-3T	TLM-1.1B	lightblue/suzume-llama-3-8B-multilingual	LM3Z-8B
Qwen/Qwen1.5-7B	QWN-7B	meta-llama/Meta-Llama-3-8B-Instruct	LMMI-8B
Qwen/Qwen1.5-7B-Chat	QWNC-7B	meta-llama/Meta-Llama-3-8B	LMM-8B
stabilityai/stablelm-2‐1_6b	STA-6B		

a The suffix (B) represents billions of parameters.

## Results

**Figure 1.**
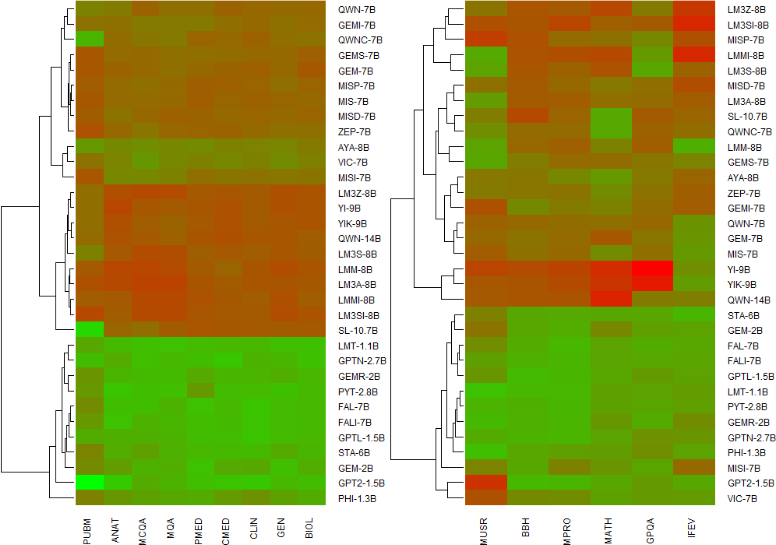
Dendrogram representing the domain-specific (DS; left) and domain non-specific (DN; right) taxonomy portraits from hierarchical clustering of scaled multivariate performance benchmark profiles of 33 medical LLMs. The magnitude of the scaled performance benchmark profiles increases from green (low) to red (high).

**Figure 2.**
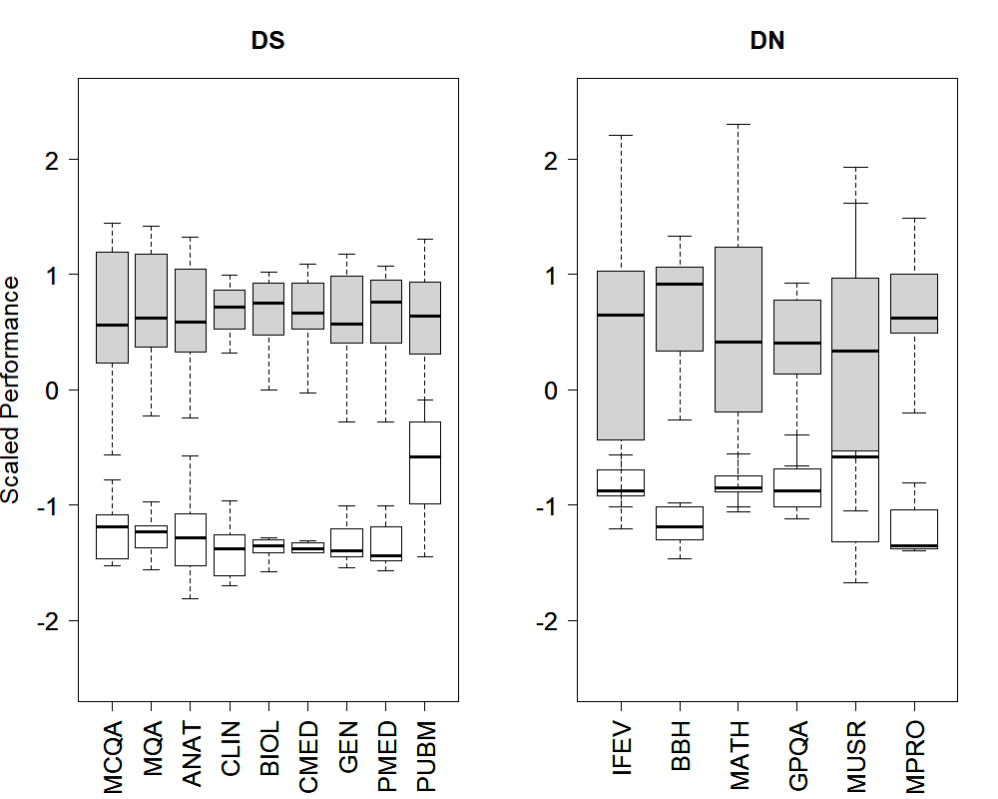
Box-whisker plots of the scaled performance benchmark profiles corresponding to high (H; gray) and low (L; white) clusters of the domain-specific (DS; left) and the domain non-specific (DN; right) taxonomies. Outliers are not shown for clarity.

The DS and DN taxonomies of the 33 medical LLMs generated by hierarchical clustering (Figure 1) revealed two well-separated clusters of high (H) and low (L) with markedly different median benchmark profiles (Figure 2). For the DS taxonomy, the H cluster comprised 22 LLMs, whereas the L cluster had 11 LLMs. The Wilcoxon rank-sum tests were used to assess statistically significant differences between these clusters across the nine benchmarks at the adjusted significance level controlling for family-wise error rate (α^*^=α/M=.01/9~.001). The corresponding *P* values were MCQA (*P*<.001), MQA (*P*<.001), ANAT (*P*<.001), CLIN (*P*<.001), BIOL (*P*<.001), CMED (*P*<.001), GEN (*P*<.001), PMED (*P*<.001), and PUBM (*P*<.001), revealing statistically significant differential changes in the nine benchmarks between the H and L clusters. LLMs in the H cluster were MISI-7B, MIS-7B, VIC-7B, LM3S-8B, QWN-14B, LM3A-8B, GEMI-7B, GEM-7B, QWN-7B, QWNC-7B, GEMS-7B, LM3SI-8B, ZEP-7B, MISD-7B, MISP-7B, AYA-8B, SL-10.7B, YIK-9B, YI-9B, LM3Z-8B, LMMI-8B, and LMM-8B, whereas those in the L cluster were PYT-2.8B, GPTN-2.7B, GEM-2B, GEMR-2B, PHI-1.3B, LMT-1.1B, STA-6B, GPTL-1.5B, GPT2-1.5B, FALI-7B, and FAL-7B. The L cluster with a relatively lower median performance profile consisted primarily of LLMs with relatively smaller numbers of parameters. While earlier studies [31,32] emphasized the impact of parameters on LLM performance, the present findings reiterated these empirical findings from a DS standpoint.

For the DN taxonomy, the H and the L clusters comprised 20 LLMs and 13 LLMs, respectively (Figure 1). The Wilcoxon rank-sum tests of differential change between the H and L clusters across the six benchmarks at the adjusted significance level controlling for family-wise error rate (α^*^=α/M=0.01/6~0.002) resulted in IFEV (*P*<.001), BBH (*P*<.001), MATH (*P*<.001), GPQA (*P*<.001), and MPRO (*P*<.001), indicating significant differential change across these benchmarks. However, the differential change for MUSR (*P*=.036) was not statistically significant. The corresponding boxplots are shown in Figure 2. LLMs in the H cluster were MIS-7B, LM3S-8B, QWN-14B, LM3A-8B, GEMI-7B, GEM-7B, QWN-7B, QWNC-7B, GEMS-7B, LM3SI-8B, ZEP-7B, MISD-7B, MISP-7B, AYA-8B, SL-10.7B, YIK-9B, YI-9B, LM3Z-8B, LMMI-8B, and LMM-8B, whereas those in the L cluster were MISI-7B, PYT-2.8B, GPTN-2.7B, VIC-7B, GEM-2B, GEMR-2B, PHI-1.3B, LMT-1.1B, STA-6B, GPTL-1.5B, GPT2-1.5B, FALI-7B, and FAL-7B. In line with earlier empirical observations, LLMs in the L cluster with relatively lower median performance benchmarks were predominantly smaller in size. There were also marked consensus in the distribution of the LLMs between the L (11 LLMs) and H (20 LLMs) clusters of the DS and DN taxonomies, revealing the robustness of the LLMs across generic as well as domain-specific tasks. The variance for a majority of the performance benchmarks was markedly higher for the H cluster as opposed to the L cluster across DS and DN taxonomies, indicating considerable heterogeneity in the performance of the LLMs in the H cluster (Figure 2).

**Figure 3.**
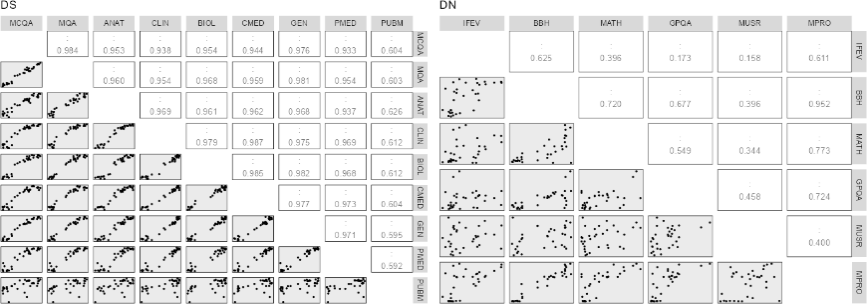
Scatter plots (gray panels) of the correlation structure between the performance benchmarks corresponding to domain-specific (DS; left) and domain non-specific (DN; right) taxonomies are shown. The pair-wise linear correlation (white panels) of the corresponding (row, column) pairs of performance benchmarks are shown in the upper triangle. The diagonals represent auto-correlation, and are hence not presented.

The Pearson correlation was used to assess statistically significant correlations between pair-wise performance benchmarks of the DS and DN taxonomies (Figure 3). The nine DS performance benchmarks resulted in 9(9‐1)/2=36 independent tests for correlation. Therefore, the adjusted significance level was chosen as (α^*^=0.01/36~0.0002) to control for the family-wise error rate. The correlation for all pairs other than PMED-PUBM (*P*=.00028>α^*^) were statistically significant (ie, *P*<α^*^). The linear trend was especially pronounced between MCQA, MQA, and MMLU for various subjects. A possible explanation is LLMs that perform well on the different medically related MMLU subject-wise benchmarks (ANAT, CLIN, BIOL, CMED, GEN, and PMED) may also perform well on comprehensive medical exams (MCQA and MQA). While the linear correlation between the MMLU subject-wise benchmarks was statistically significant, the scatter plots revealed instances of sparse distribution of points along the linear trend line for some of these pairs (eg, BIOL and PMED), challenging reliable correlation estimates (Figure 3). The clustering of points about the linear trend line may in fact align with earlier observations of statistical differences (Figure 2) between two broad groups of LLMs within the DS taxonomy (Figures1  2). The magnitude of performance benchmark (PUBM), which assesses the LLMs ability to comprehend and reason biomedical literature, did not exhibit a strong correlation with others, as reflected by lack of a clear linear trend in the scatter plots (Figure 3). In contrast to DS taxonomy, the correlation structures between the performance benchmarks was noisy in the case of DN taxonomy, as reflected by the scatter plots in Figure 3. The six DS performance benchmarks resulted in 6(6‐1)/2=15 independent tests for correlation. Therefore, the adjusted significance level was chosen as (α^*^=0.01/15~0.0007) to control for the family-wise error rate. While there were instances of DN performance benchmark pairs with significant correlation, the redundancy was markedly lower than that observed in the case of DS taxonomy. Pairs that did not exhibit significant correlation (*P*<α^*^) included IFEV-MATH (*P*=.022), IFEV-GPQA (*P*=.336), IFEV-MUSR (*P*=.379), BBH-MUSR (*P*=.022), MATH-MUSR (*P*=0.049), GPQA-MUSR (*P*=.007), and MUSR-MPRO (*P*=.021). Since the correlation structure across DN performance benchmarks was much lower than that of DS performance benchmarks, DN performance benchmarks may assess the LLMs complementary characteristics of the LLM.

## Discussion

The present study investigated taxonomies of LLMs whose DS and DN performance benchmarks were available from Open MedLLM and Open LLM initiatives at Hugging Face. As noted, LLM ranks estimated from aggregated benchmarks have been featured by leaderboards, impacting their adoption. However, similarity in these aggregated univariate scores and ranks may not necessarily imply similarity in the underlying multivariate performance profiles, making the study relevant. The DS and DN taxonomies revealed inherent subgroups and two broad clusters with statistically significant differences in performance. Overlap of the cluster members between the DS and DN taxonomies also indicated robustness of these LLMs across diverse tasks. As with some of the earlier empirical studies on LLM scaling laws, clusters with lower performance consisted predominantly of LLMs with relatively smaller size. The results also revealed redundancies in the performance benchmarks that was especially pronounced in the case of DS performance benchmarks.

Practical relevance of these taxonomies include their ability to assist in selecting LLMs with comparable performance while controlling costs. This is especially critical in low-profit margins industry such as health care that is in its initial phases of adopting AI tools for improved efficiency and outcomes. Taxonomies can also assist in choosing a combination of LLMs, perhaps across distinct clusters with diverse performance characteristics [33,34] as opposed to a single LLM. However, unlike performance benchmarks, the lack of transparency with regards to costs and LLM economics [35] discourages concerted analysis of costs and performance benchmarks. Moreover, several factors can impact LLM economics including digital readiness, infrastructure, workforce, and cloud-based implementations [9]. While faithful cost estimation can be challenging, economics of LLM training, fine-tuning, and inference is generally agreed to be proportional to the LLM size. Thus, the size of the LLM can serve as a surrogate for costs. The DS and DN taxonomies revealed considerable consensus and two broad clusters (H, L). For the H cluster, LLMs QWN-14B and YIK-9B were proximal across the DS and DN taxonomy. However, the size of QWN-14B (~14 billion parameters) was considerably larger than that of YIK-9B (~9 billion parameters). Based on the DS and DN taxonomies, YIK-9B is preferred over QWN-14B. On a related note, the L cluster also comprised LLMs of markedly different sizes with comparable performance. For instance, FALI-7B (~7 billion parameters) was proximal to GPTL-1.5B (~2.8 billion parameters) recommending GPTL-1.5B over FALI-7B. Pair-wise correlation profiles revealed marked association between the DS performance benchmarks. While performance benchmarks are expected to ideally interrogate complementary characteristics of an LLM, the presence of correlations indicated inherent redundancies between the DS benchmarks. As LLM ranks are generally estimated as the weighted average of the performance benchmarks, eliminating redundancies may be critical for generating unbiased rank estimates. Eliminating redundant benchmarks can also assist in minimizing the overall evaluation cost [36].

There are several limitations of the present study. The study focused primarily on medium-sized LLMs (n=33) with around tens of billions of parameters. Generating comprehensive taxonomy portraits with a larger pool of LLMs spanning a wider range of sizes (large, ~100 billion parameters; medium, ~10 billion parameters; and small, ~millions of parameters) can reveal universal patterns characteristic of medical LLMs. Such an analysis may also assist in selecting LLM ensembles with varying sizes and complementary performance benchmark profiles, as opposed to the popular practice of selecting a single LLM. While the present study focused on standardized performance benchmarks, it may have limited usefulness in assessing capabilities [37] such as summarization, used routinely by health care conversational AI agents (eg, Chatbots and Ambient Listening Tools). The non-deterministic nature of the LLM response can also pose challenges in assessing such summaries, especially when these tools are deployed in clinical workflows [38]. While performance benchmarks used in generating the DS and DN taxonomies interrogate certain unique characteristics of the LLMs, they are by no means exhaustive. The rapid evolution of LLMs and their health care applications might demand incorporating novel benchmarks. Assessment may also explore relative benchmarking strategies, where characteristics, such as factual accuracy of information, time to retrieve information, and ease of use, are compared to tools that are currently in place using randomized controlled designs. While the size of the LLMs were used as surrogates for costs in the present study, e enhanced transparency on LLM economics across training, fine-tuning, and inference could assist in tailored recommendations for strategic decision making in health care settings. As noted earlier, several factors can dictate the economics of implementation, deployment, and operationalization of LLMs in health care workflows. This includes digital and analytics maturity, infrastructure, workforce across a spectrum of areas, choice of the LLM onboarding pathways, and the needs of the health system. While the focus of the present study has been primarily on open-source LLMs, enhanced transparency of closed-source LLMs can facilitate unbiased comparisons for equitable and strategic adoption of these tools across health care enterprises.

## Abbreviations

DN: domain non-specific
DS: domain-specific
H: high
L: low
LLM: large language model
